# From Self-Esteem to Symptoms: A Potential Role for Difficulties Accessing Internal States and Body-Checking Behavior in Disordered Eating Patterns

**DOI:** 10.3390/bs16030434

**Published:** 2026-03-17

**Authors:** Diana Arbich, Daniela Kaplan, Reuven Dar

**Affiliations:** The School of Psychological Sciences, Tel Aviv University, Tel Aviv 69978, Israel

**Keywords:** body image, internal states, proxies, self-esteem, body-checking, eating disorders, disordered eating patterns, mediation

## Abstract

Drawing on the Seeking Proxies for Internal States (SPIS) model and the concept of Difficulties in Accessing Internal States (DAIS), the present study examined the statistical associations among self-esteem, DAIS, body-checking, and disordered eating patterns (DEP). Within the SPIS framework, self-esteem is conceptualized as an evaluative internal state that may be appraised through externally observable proxies, such as body appearance. Cross-sectional data were collected from 200 adults recruited through Prolific Academic. Hayes’ PROCESS macro was used to test simple and serial mediation models examining whether DAIS and body-checking statistically account for associations between state self-esteem and DEP. Lower self-esteem was associated with higher DEP. Both DAIS and body-checking statistically accounted for portions of this association in simple mediation models. In a serial mediation model, the fully sequential pathway (self-esteem → DAIS → body-checking → DEP) remained statistically significant after accounting for shared variance among mediators. Given the cross-sectional design, these findings cannot establish temporal or causal relationships, but the observed pattern of associations is compatible with the proposed conceptual process. Additionally, our findings are based on a nonclinical sample and reflect variability in subclinical eating pathology. Implications for extending the SPIS framework to dimensional eating-related phenomena are discussed.

## 1. Introduction

Research on the psychological processes underlying eating disorders, or broadly, disordered eating patterns (DEP), has identified self-esteem and body-checking as core elements (Walker et al., 2018; Nikodijevic et al., 2018; Krauss et al., 2023). The link between self-esteem, body-checking, and DEP, however, has not been fully elucidated. In this study, we suggest that incorporating concepts from the Seeking Proxies for Internal States (SPIS) model, originally conceived for obsessive-compulsive disorder (OCD), might illuminate mechanisms behind these relationships. Specifically, we propose that concepts from the SPIS model can account for the link between self-esteem and body-checking in individuals with DEP.

### 1.1. The SPIS Model and the Concept of DAIS

According to the SPIS model, many OCD symptoms stem from attenuated access to internal states (for reviews of the model see Dar et al., 2021; Liberman et al., 2023). The model defines internal states as states within a person’s system to which they have privileged access, including physiological (e.g., hunger, proprioception), cognitive (e.g., memory, a sense of understanding), emotional (e.g., love, satisfaction), or other “private” states such as preferences and motivations. According to the SPIS model, when a question regarding an internal state arises (e.g., “Do I love my partner?”) and the person encounters difficulties accessing that internal state, they might seek an external proxy to answer the question (e.g., checking how often they call their partner).

A core concept underlying the SPIS model is difficulties in accessing internal states (henceforth DAIS; Liberman & Dar, 2018). While DAIS was originally considered an underlying feature of OCD, it also characterizes other conditions, including alexithymia (difficulties in identifying and describing one’s emotions; Hemming et al., 2019) and interoceptive deficits (difficulties in detecting and processing internal bodily sensations; Craig, 2003). These frequently co-occurring deficits have both been suggested as mechanisms involved in a multitude of psychopathologies (Kick et al., 2024; Brewer et al., 2016, 2021; Gaggero et al., 2021; Herbert et al., 2011), supporting the potential relevance of the DAIS concept beyond OCD.

According to the SPIS model, DAIS is associated with seeking and reliance on compensatory proxies—external indicators of internal states that the individual perceives as more easily discernible. This notion appears highly relevant to disordered eating, as both clinical and non-clinical individuals with DEP show deficits in identifying and communicating both physical and emotional states (Simonsen et al., 2020; Nandrino et al., 2012; De Berardis et al., 2007; Nowakowski et al., 2013; Sasai et al., 2010). Both interoceptive deficits and alexithymia are consistently found in clinical and non-clinical individuals with DEP, and poor interoceptive awareness is even identified as a risk factor for their development (Jenkinson et al., 2018; Johnson, 2026; Leon et al., 1995; Perkins et al., 2021; Westwood et al., 2017). Many studies also find biological underpinnings for interoceptive differences, including neurological abnormalities in structure and the connectivity of interoceptive circuits (Frank et al., 2023; Klabunde et al., 2017). These findings point to the relevance of considering alexithymia and interoceptive deficits in conjunction with eating disorders, and specifically of exploring a possible role for DAIS in DEP. In addition, the frequent occurrence of OCD-related characteristics, such as perfectionism and obsessionality, in eating disorders (Meier et al., 2020; Robinson & Wade, 2021; Georgantopoulos et al., 2020) warrants further investigation in this direction.

### 1.2. Self-Esteem in Eating Disorders

Several theoretical approaches identify self-esteem as a central vulnerability factor in the development and maintenance of eating pathology (Biney et al., 2022; Bhar & Kyrios, 2016; Kästner et al., 2019; Yelsma, 1995). Cognitive-behavioral models propose that, in eating disorders, self-worth becomes disproportionately defined by weight, shape, and control over eating (Fairburn et al., 2003). Within this framework, individuals with low or fragile self-esteem may pursue achievement in the overvalued domain of body control in an attempt to regulate their sense of worth.

The self-schema model similarly suggests that individuals may develop dominant body-weight self-schemas that guide attention, evaluation, and behavior (Markus, 1977; Markus et al., 1987). When self-evaluation becomes heavily anchored in body-related domains, behaviors such as dieting and body-checking may acquire heightened personal significance (Rohde et al., 2015). Importantly, self-esteem can be conceptualized at multiple levels, including as a relatively stable trait and as a fluctuating evaluative state. The present study focuses on state self-esteem—an experientially accessible, momentary sense of self-worth (Heatherton & Polivy, 1991; Webster et al., 2022). State self-esteem represents a subjective evaluative internal experience that individuals can access and appraise, even if imperfectly.

With this conceptualization, the SPIS scaffolding becomes relevant. Body-checking behaviors may function as attempts to monitor an external indicator (body appearance) that is perceived as relevant to one’s self-worth. When self-esteem is fragile or uncertain, individuals may rely more heavily on observable bodily cues to infer personal value. This perspective opens the possibility that body-checking may operate not merely as a behavioral symptom, but as part of a broader strategy for evaluating self-worth.

In extending the SPIS framework to this domain, we conceptualize self-esteem as an evaluative internal state rather than as an abstract trait construct. In the SPIS model, internal states are defined as subjectively accessible experiences to which individuals have privileged access (Dar et al., 2021). We propose that the subjective sense of self-worth qualifies as such an evaluative internal state. The “question” within the SPIS framework (e.g., “Am I a worthwhile person?”) refers to uncertainty about this evaluative state. Difficulties in accessing internal states (DAIS), in this context, reflect difficulty in confidently appraising one’s internal evaluative experience. Importantly, we do not propose that DAIS lowers self-esteem. Rather, when access to evaluative internal states is diminished or uncertain, individuals may rely more heavily on externally observable proxies—such as body appearance—to infer their self-worth. In fact, lower self-esteem and body-esteem have been correlated with both higher alexithymia and disordered eating in a non-clinical sample (Sasai et al., 2010).

In the context of low self-esteem, the “answers” obtained by body-checking tend to be interpreted negatively. This negative bias may stem from psychological characteristics that are relatively prevalent in those with eating disorders (Fairburn et al., 2008), such as perfectionism (Galloway et al., 2022), cognitive inflexibility, and obsessionality (Call et al., 2017). Consequently, frequent body-checking may reinforce body dissatisfaction in prone individuals, undermining the behavior’s intended outcome and further directing excessive attention towards estimates of size or shape to be interpreted through a self-criticizing lens (Williamson et al., 1999; Reas et al., 2002; Bardone-Cone et al., 2020). To control these markers of self-worth, an individual may employ eating patterns aimed at directly influencing the markers examined by body-checking (De Berardis et al., 2007). Due to the aforementioned negative evaluation bias, a loop is completed whereby DEP are promoted by the selective interpretation of body-related data gathered by body-checking.

### 1.3. The Current Study

The present study integrates principles from the SPIS model with established theories of eating pathology to examine whether the associations among self-esteem, difficulties accessing internal states (DAISs), body-checking, and disordered eating patterns (DEPs) are consistent with a theoretically derived ordering.

Specifically, we propose the following conceptual sequence: lower state self-esteem may be associated with greater difficulty accessing internal evaluative states (DAISs). When internal evaluative clarity is reduced, individuals may rely more heavily on externally observable indicators of worth, such as body appearance, and engage in body-checking behaviors to appraise these proxies. Increased reliance on such appearance-based evaluation strategies may, in turn, be associated with greater disordered eating tendencies.

Given the cross-sectional nature of the data, the present analyses do not test temporal processes or establish causal pathways. Rather, we examine whether the observed pattern of statistical associations is compatible with this theoretically informed ordering. Accordingly, we tested (a) whether DAIS statistically accounts for part of the association between self-esteem and DEP, (b) whether body-checking does so, and (c) whether a serial mediation model in which DAIS and body-checking are positioned sequentially is consistent with the data. This study represents an initial examination of whether concepts derived from the SPIS framework may help illuminate psychological processes associated with dimensional disordered eating tendencies in a nonclinical sample.

## 2. Materials and Methods

### 2.1. Participants

Two hundred and two non-clinical English-speaking adults were recruited through the *Prolific Academic* website (https://prolific.co; accessed 22 June 2022). Two participants were excluded, one due to a reported psychiatric diagnosis and a second due to an unreasonably short response time. The final sample comprised 200 participants: 81 males (*M_age_* = 42.02 years, *SD* = 13.95, *R* = 22–79 years) and 119 females (*M_age_* = 39.17 years, *SD* = 14.19, *R* = 18–80 years), who were residents of either the United States or the United Kingdom. All participants denied having a current or past diagnosis of a psychiatric disorder. Participants read a brief description of the study and marked their agreement to participate before receiving the study materials. Participants received approximately £8.30 per hour as compensation for completing the study. The study protocol was approved by the Institutional Review Board (IRB) of Tel Aviv University.

### 2.2. Materials

To assess DAIS, we combined items from three established scales that evaluate specific aspects of the construct. Specifically, two of the scales assess alexithymia, which, as discussed above, involves difficulty in accessing emotions. The third scale, in contrast, assesses the use of proxies to compensate for attenuated access to a variety of internal states. We termed the combined measure *Difficulties in Accessing Internal States-Composite Scale (DAIS-CS)*. The composite scale comprised items from the following scales:

*1. The Toronto Alexithymia Scale* (TAS-20; Bagby et al., 1994) assesses people’s ability to access and label their emotions. The scale has 20 items rated on a 5-point scale from “completely disagree” to “completely agree.” We used two TAS-20 subscales that are relevant to DAIS: difficulty identifying feelings (5 items, e.g., “I am often confused about what emotion I am feeling”); and difficulty describing feelings (7 items, e.g., “It is difficult for me to find the right words for my feelings”). The third subscale, externally oriented thinking (8 items, e.g., “I prefer to analyze problems rather than just describe them”), is conceptually unrelated to DAIS and was therefore not included in the DAIS-CS. The modified version of the TAS-20 had a Cronbach Alpha of 0.92.

*2. The State–Emotion Similarity Scale* (Brewer et al., 2016) uses a different approach to assess alexithymia, by measuring the similarity respondents experience between emotions (e.g., happiness, disgust) and internal states (e.g., feeling cold, hunger). For instance: “How similar are your personal experiences of being hot and feeling happy? (1 = Not at all; 7 = Very similar)”. The original scale included 72 items, combining twelve internal states with six emotions, and demonstrated excellent internal consistency (α = 0.96). However, as only some combinations of emotions and internal states were judged as at least somewhat similar in the original study, even by participants with alexithymia (Brewer et al., 2016), the present study retained 20 relevant combinations of states (feeling cold, nausea, hunger, physical fatigue, shortness of breath, and racing heartbeat) and emotions (sadness, disgust, anger, and fear). The modified version of the State–Emotion Similarity Scale had a Cronbach Alpha of 0.94.

*3. The Seeking Proxies for Internal States Inventory* (SPISI; Liberman & Dar, 2018) assesses the degree to which responders seek and depend on proxies to interpret internal states in their daily lives. Some items address specific internal states and proxies (e.g., “To know how hungry I am, I consider what and when I’ve eaten today”); others investigate general tendencies (e.g., “I look for rules that would tell me what I’m supposed to do”). Participants are asked to evaluate the extent to which each statement applies to them, on a 5-point scale from “Not at all” to “Very much.” Cronbach alpha demonstrated strong internal consistency in both an Israeli and a Dutch sample (α = 0.86–0.87). In the present study, the SPISI had a Cronbach Alpha of 0.90.

Although the three component measures assess conceptually distinct facets—emotion identification, state–emotion similarity, and proxy-seeking—they were selected as each reflects a theoretically related aspect of diminished access to internal states. We therefore conceptualized these measures as complementary indicators of a broader higher-order construct rather than as interchangeable constructs. The composite was intended to capture shared variance across these facets while acknowledging their conceptual distinctiveness.

To assess the psychometric properties of the new DAIS-CS, we submitted the three scales detailed above to a principal component analysis (PCA). Analysis was conducted on JASP (Version 0.95.4), using parallel analysis with Promax rotation. The three scales were moderately to highly inter-correlated (r’s = 0.44–0.65), and the PCA resulted in a single factor on which the three scales had very high loadings (0.75–0.87). The single factor had an eigenvalue of 2.05 and explained 68.5% of the variance in the three scales. Finally, a simple sum of the three measures (normalized) scores, which is the version we used in the present study, correlated with the factor score almost perfectly (r (200) = 0.99). The Cronbach’s Alpha of the DAIS-CS was 0.95, indicating a very high internal consistency.

*Additional Measures*

In addition to the *DAIS-CS*, participants responded to the three measures described below. Participants also provided basic demographic information as well as their height and weight, which were used to calculate BMI (weight/height^2^ (kg/m^2^).

The *Body Checking Questionnaire* (*BCQ*; Reas et al., 2002; Calugi et al., 2006) evaluates the self-reported frequency of body-checking behaviors using 23 items, each followed by a 5-point Likert scale (1 = “*never*”; 5 = “*very often*”). The BCQ has demonstrated good convergent validity with measures of eating pathology in clinical (Shafran et al., 2004) and non-clinical samples (Reas et al., 2009; Lydecker et al., 2014; White et al., 2015). As the current study focuses on global appearance-based comparison tendencies, including all subscales could have obscured the relationships we sought to test by adding body-part–specific variance unrelated to our theoretical focus. We therefore used only the overall appearance (OA) subscale, comprising 10 items, that had a Cronbach’s Alpha of 0.89. 

The *Eating Attitude Test-26 (EAT-26)* is widely used to identify predisposition to eating disorders and has demonstrated validity in clinical and non-clinical samples (Garner et al., 1982; Lee et al., 2002; Mintz & O’Halloran, 2000). Twenty-six items comprise three subscales: (1) Dieting, (2) Bulimia and Food Preoccupation, and (3) Oral Control. A 6-point Likert scale follows each item (1 = “*never*”; 6 = “*always*”). In the present study, Cronbach’s Alpha of the EAT-26 was 0.91.

The *Six-Item State Self-Esteem Scale* (*SESS-6*; Webster et al., 2022), derived from the SESS-20 scale (Heatherton & Polivy, 1991), is divided into three components of self-esteem: social, appearance, and performance. Items are rated on a 7-point Likert scale (1 = “*Strongly disagree*”; 7 = “*Strongly agree*”). We used a reversed scoring method to maintain response uniformity with the other scales used in this study, such that a higher SESS-6 score represents lower self-esteem. In the present study, Cronbach’s Alpha of the SESS-6 was 0.87.

### 2.3. Procedure

Participants provided personal data (age, gender, height, weight) and then answered the questionnaires in the following order: DAIS-CS, BCQ, SESS-6, and EAT-26. To ensure data quality, we included two Instructional Manipulation Checks (IMCs), which instructed the participants not to answer two of the questions. Participants who failed to follow these instructions were excluded and notified accordingly (Oppenheimer et al., 2009; Paas et al., 2018).

### 2.4. Data Analysis

**Figure 1:**
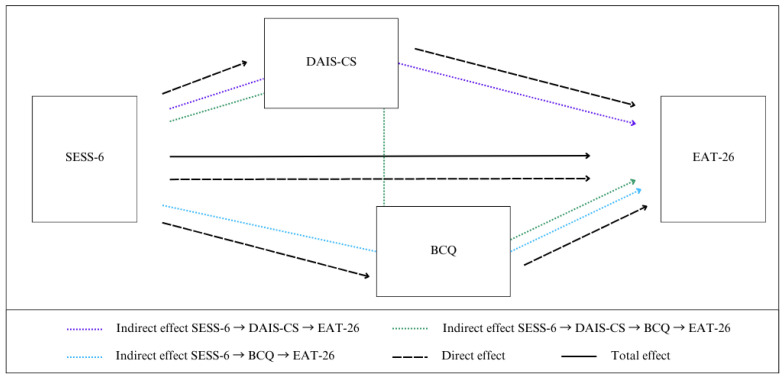
Schema illustrating the effect of self-esteem on disordered eating patterns mediated by difficulties in accessing internal states and body-checking.

Relationships between continuous variables were examined using Pearson’s correlation coefficients. This was followed by two simple mediation analyses testing the separate contributions of each of the mediators (DAIS and body-checking) to the link between self-esteem and DEP. Finally, our main analysis tested the hypothesized sequence of mediation involving both mediators, i.e., self-esteem → DAIS → body-checking → DEP. Figure 1 presents a schema of these analyses conducted in the study.

Statistical analysis was conducted using SPSS software (IBM Corp, 2020, 2021). Following Hayes’s guidelines (Hayes, 2013), the SPSS PROCESS macros v. 3.4, followed by v.4.0, were used to test mediation effects. All mediation analyses were adjusted for the covariates of age, BMI, and gender. The simple (Model 4) and serial multiple (Model 6; Hayes, 2018) mediation analyses were based on 5000 bootstrap samples using 95% confidence intervals (CIs). Bootstrapping was used to compensate for any power lost using the PROCESS macro. Effect weights were reported as regression unstandardized coefficients (*B*; Baguley, 2009). If CIs did not contain zero, indirect effects were considered significant, indicating a mediated effect (Hayes, 2018). As PROCESS does not produce a *p*-value for indirect effects, the mediated effects’ statistical significance was confirmed with Sobel’s test (Sobel, 1982; Preacher & Hayes, 2008).

## 3. Results

Model assumptions were evaluated prior to analysis; details are reported in Supplementary Materials S1.

Table 1 displays descriptive statistics and bivariate correlation results. The DAIS-CS correlated positively with SESS-6, BCQ (OA), and EAT-26. BCQ was also positively correlated with SESS-6 and EAT-26. Lastly, SESS-6 correlated positively with EAT-26. These results support our initial hypotheses regarding the associations between these factors.

### 3.1. DAIS as a Mediator of the Effect That Self-Esteem Exerts on DEP

The results of a simple mediation analysis examining whether DAIS-CS statistically accounted for part of the association between self-esteem and DEP are presented in Table 2. As detailed, the estimated paths were statistically significant as confirmed by Sobel’s test (z = 2.38, *p* = 0.017). Regarding model fit, the direct effect model was statistically significant, *F*(5,194) = 10.55, *p* < 0.001, with predictors accounting for approximately 21% of the variance in eating pathology (*R*^2^ = 0.21, Adjusted *R*^2^ = 0.19). The multiple correlation coefficient was *R* = 0.46, suggesting a moderate association between predicted and observed values.

### 3.2. Body-Checking as a Mediator of the Effect That Self-Esteem Exerts on DEP

We conducted a simple mediation analysis to examine whether body-checking statistically accounted for part of the association between self-esteem and DEP (Table 3). The bootstrap confidence interval for the indirect pathway did not include zero, suggesting that body-checking was statistically associated with variation in DEP in this model (Sobel’s test: z = 3.63, *p* < 0.001). The overall regression model was statistically significant, *F*(5,194) = 19.51, *p* < 0.001, with predictors accounting for approximately 33% of the variance in DEP (*R*^2^ = 0.33, Adjusted *R*^2^ = 0.31). The multiple correlation coefficient (*R* = 0.58) suggests a moderate to strong association between predicted and observed values.

### 3.3. Main Analysis: DAIS and Body-Checking as Serial Predictors in the Association Between Self-Esteem and DEP

To examine whether the pattern of associations was consistent with the proposed sequence, we estimated a serial mediation model whereby DAIS-CS and body-checking were positioned sequentially between self-esteem and DEP (Table 4). The overall regression model was statistically significant, *F*(6,193) = 16.26, *p* < 0.001, with predictors accounting for approximately 34% of the variance in EAT-26 scores (*R*^2^ = 0.34, Adjusted *R*^2^ = 0.32). The multiple correlation coefficient (*R* = 0.58) indicates a moderate to strong association between predicted and observed values.

The bootstrap confidence interval for the total indirect pathway did not include zero, suggesting that, taken together, the mediators statistically accounted for some of the association between self-esteem and DEPs. Importantly, the fully sequential indirect pathway (self-esteem → DAIS-CS → body-checking → DEP) also yielded a confidence interval that excluded zero, indicating that this ordered pathway was statistically compatible with the data.

In contrast, when each mediator was examined within the serial model while accounting for the other, the indirect pathways involving only DAIS-CS or only body-checking did not yield confidence intervals excluding zero. This pattern indicates that, within this statistical model, the fully sequential pathway accounted for the largest portion of the indirect association. Given the cross-sectional design, however, this finding should be interpreted as reflecting statistical positioning within the model rather than evidence of temporal or causal processes. Alternative explanations, including shared variance or suppression effects among mediators, remain possible.

## 4. Discussion

The present study examined whether the pattern of associations among state self-esteem, difficulties accessing internal states (DAIS), body-checking, and disordered eating patterns (DEP) is statistically consistent with a theoretically derived ordering informed by the Seeking Proxies for Internal States (SPIS) framework (Dar et al., 2021; Liberman et al., 2023). Although the cross-sectional design does not permit conclusions regarding temporal or causal processes, the observed pattern of associations aligns with the proposed conceptual model.

Consistent with extensive prior research, lower self-esteem was associated with greater disordered eating tendencies. This finding replicates well-established evidence identifying fragile or diminished self-worth as a vulnerability factor across eating-related phenomena (Fairburn et al., 2003; Bardone-Cone et al., 2020; Krauss et al., 2023; Sahlan et al., 2021). Cognitive-behavioral models propose that in eating disorders, self-worth becomes disproportionately defined by weight, shape, and eating control (Fairburn et al., 1999, 2003). The present findings are consistent with this literature in demonstrating a robust association between evaluative self-experience and eating-related symptoms within a general population sample.

In the simple mediation models, both DAIS and body-checking statistically accounted for portions of the association between self-esteem and DEP. These findings suggest that diminished clarity regarding internal states and increased reliance on appearance-based monitoring are meaningfully associated with eating-related symptomatology. Prior work has linked alexithymia and interoceptive deficits to eating disorder symptoms (Jenkinson et al., 2018; Leon et al., 1995; Nowakowski et al., 2013; Nandrino et al., 2012; Perkins et al., 2021), and body-checking has been identified as both an etiological and maintaining factor in eating disorders and non-clinical eating disorder symptoms (De Berardis et al., 2007; Reas et al., 2002; Nikodijevic et al., 2018). The present findings integrate these strands of research within a unified statistical framework.

In the serial mediation model, only the fully sequential indirect pathway (self-esteem → DAIS → body-checking → DEP) yielded a confidence interval excluding zero when both mediators were included simultaneously. This statistical pattern is compatible with the proposed conceptual ordering. However, because all variables were assessed concurrently, this ordering reflects theoretical specification rather than temporal sequencing. Alternative interpretations—including shared variance among mediators, suppression effects, or bidirectional relationships—remain possible. Longitudinal or experimental designs would be required to test whether the proposed sequence unfolds over time.

A central conceptual contribution of the present study lies in extending the SPIS framework beyond obsessive–compulsive phenomena to dimensional eating-related tendencies. Within this extension, self-esteem is conceptualized as an evaluative internal state—namely, a subjectively experienced sense of self-worth that individuals can access, appraise, and question (Heatherton & Polivy, 1991; Webster et al., 2022). The “question” in the SPIS framework (e.g., “Am I a worthwhile person?”) reflects uncertainty about this evaluative state. DAIS, in this context, refers to difficulty confidently accessing or appraising internal evaluative experiences. Importantly, we do not propose that DAIS lowers self-esteem. Rather, when access to internal evaluative states is diminished or uncertain, individuals may rely more heavily on externally observable proxies—such as body appearance—to infer their worth. Body-checking may then function as a behavioral strategy for gathering information about this proxy. The present findings are statistically consistent with this interpretation.

These results can also be considered in relation to self-objectification theory (Fredrickson & Roberts, 1997), which posits that habitual body monitoring may diminish interoceptive awareness. Although our serial model positions DAIS before body-checking in the tested ordering, the cross-sectional design precludes firm conclusions regarding directionality. It remains plausible that reciprocal or mutually reinforcing processes operate between internal state awareness and external body monitoring. The present findings highlight the close statistical association among these constructs and underscore the need for temporally sensitive research designs.

Additionally, emerging research on self-compassion provides converging evidence relevant to the present framework. Self-compassion has been associated with reduced body-checking and eating pathology in non-clinical samples (Huellemann & Calogero, 2020; Matera et al., 2024; Di Gesto et al., 2023). Within the current conceptualization, self-compassion may buffer against reliance on appearance-based proxies when self-worth is uncertain. Future studies could examine whether self-compassion moderates associations among self-esteem, DAIS, and body-checking.

**Figure 2:**
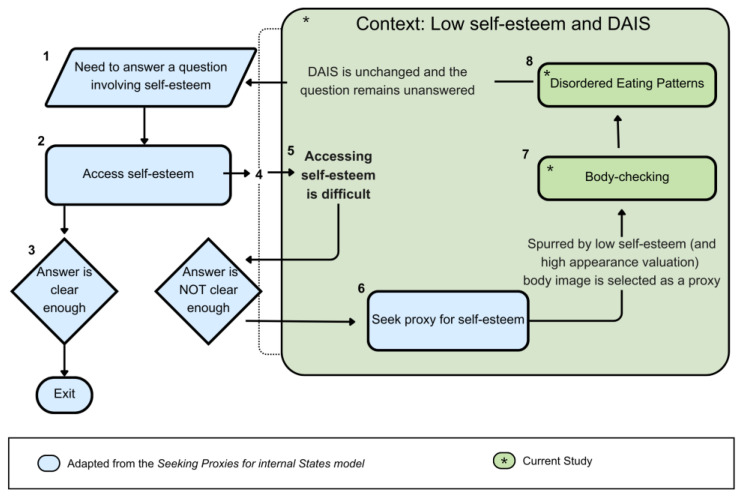
Conceptual adaptation of the SPIS model to disordered eating patterns.

To illustrate the conceptual integration proposed in this study, Figure 2 presents a schematic adaptation of the SPIS framework to disordered eating patterns. In this representation, self-esteem is conceptualized as an evaluative internal state that may be appraised through externally observable proxies when access to internal evaluative experience is diminished. The figure depicts the theorized ordering among self-esteem, DAIS, body-checking, and DEP as a conceptual model derived from existing theoretical perspectives (Dar et al., 2021; Fairburn et al., 2003). Importantly, the diagram reflects theoretical specification rather than empirically established temporal sequencing. The present cross-sectional findings are statistically consistent with elements of this model but do not confirm the directional processes illustrated. An expanded explanation of the theoretical model, including the numerical indicators (corresponding to the conceptual sequence), can be found in Supplementary Materials S2.

The relatively low mean EAT-26 score in the present sample indicates that the findings reflect variability in subclinical disordered eating tendencies rather than diagnosed eating disorders. Accordingly, the proposed model pertains to dimensional eating pathology within the general population. Subclinical and clinical eating phenomena often share underlying psychological mechanisms (Mintz & O’Halloran, 2000; Maïano et al., 2013), yet generalization to clinical populations should be made cautiously. Replication in samples with clinically significant eating pathology will be necessary to determine the model’s applicability in treatment settings.

From a developmental perspective, early caregiving environments characterized by emotional inconsistency, autonomy restriction, or trauma exposure have been linked to both fragile self-esteem and alexithymia (Kick et al., 2024; Monteleone et al., 2019). Integrating such developmental risk models with the present framework may clarify distal pathways through which vulnerabilities in self-evaluation and internal state awareness emerge. Future longitudinal research incorporating relational and developmental variables could further refine and test the proposed model.

Several limitations should be acknowledged. First, the cross-sectional design precludes conclusions regarding temporal precedence or causal mediation. The ordering of variables was theoretically specified rather than empirically sequenced. Second, although the DAIS composite demonstrated strong internal coherence in the present sample, its constituent components reflect conceptually distinct facets of internal state access. Aggregating these dimensions may obscure potentially differential associations with eating pathology. Future research should examine these facets separately and validate the composite in independent samples. Third, reliance on self-report measures introduces the possibility of reporting biases. Finally, additional constructs relevant to the proposed model—such as perfectionism, internalized appearance ideals, or obsessionality (Meier et al., 2020; Robinson & Wade, 2021)—were not directly assessed.

Despite these limitations, the present findings offer preliminary support for considering DAIS and body-checking within a unified conceptual framework linking evaluative self-experience and eating-related tendencies. If supported by future longitudinal and experimental research, this perspective may enrich understanding of how reliance on externally observable proxies intersects with eating-related symptomatology and inform more targeted prevention and intervention efforts.

## 5. Conclusions

The results of this preliminary study suggest that the Seeking Proxies for Internal States (SPIS) model can be adapted to the field of disordered eating patterns. Our findings are consistent with a model in which the association between self-esteem and disordered eating is represented by a sequential pathway involving difficulties accessing internal states and body-checking. If supported by future research, the proposed model may enrich our understanding of the mechanisms behind disordered eating patterns and inform targeted interventions.

## Figures and Tables

**Table 1:** Descriptive statistics and Pearson’s correlation analysis of demographic data, self-esteem, difficulties in accessing internal states, body-checking behaviors, and disordered eating patterns.

	M	SD	1	2	3	4	5	6
Age	40.33	14.13	1					
BMI	26.89	7.26	0.04	1				
SESS-6	2.74	1.41	−0.25 ***	0.16 *	1			
DAIS-CS	15.98	7.85	−0.42 ***	0.09	0.56 ***	1		
BCQ	3.05	2.13	−0.32 ***	0.10	0.39 ***	0.51 ***	1	
**EAT-26**	**7.27**	**3.95**	−0.22 **	0.15 *	0.38 ***	0.38 ***	0.54 ***	**1**

Note. * *p* < 0.05, ** *p* < 0.01, *** *p* < 0.001; M = mean; SD = standard deviation; BMI = body mass index; SESS-6 = Six-Item State Self-Esteem scale; DAIS-CS = Difficulties in Accessing Internal States—Composite Scale; BCQ = Body Checking Questionnaire (Overall Appearance subfactor); EAT-26 = The Eating Attitudes Test-26. Results in bold indicate Pearson’s correlation between all factors and the EAT-26, supporting the link between these measures within our sample.

**Table 2:** Simple mediation analysis of the effect that self-esteem exerts on disordered eating patterns directly or via difficulties in accessing internal states (SESS-6 → DAIS-CS → EAT-26).

Effect ^a^, Variables	B	se	LLCI	ULCI	*p*-Value
Total effect (SESS-6 → EAT-26)	0.87	0.19	0.49	1.25	<0.001
Direct effect (SESS-6 → EAT-26)	0.60	0.22	0.18	1.03	0.006
Indirect effect (SESS-6 → DAIS-CS → EAT-26)	0.27	0.12	0.03	0.52	^b^
Direct effect (SESS-6 → DAIS-CS)	2.63	0.33	1.98	3.27	<0.001
Direct effect (DAIS-CS → EAT-26)	0.10	0.04	0.02	0.18	0.014

^a^ Adjusted for age, BMI, and gender covariates. ^b^ No *p*-value is calculated for the indirect effect. The indirect effect is deemed significant because the confidence interval does not include zero. Sobel’s test further confirmed the statistical significance of this indirect effect. *Note.* Based on 5000 bootstrap samples, total, direct, and indirect association of self-esteem (the predictor) and disordered eating patterns (the outcome) via the mediator (DAIS-CS) were evaluated. B = unstandardized regression coefficient; se = standard error; LLCI = lower limit of confidence interval; ULCI = upper limit of confidence interval.

**Table 3:** Simple mediation analysis of the effect that self-esteem exerts on disordered eating patterns directly or via body-checking behaviors (SESS-6 → BCQ → EAT-26).

Effect ^a^, Variables	B	se	LLCI	ULCI	*p*-Value
Total effect (SESS-6 → EAT-26)	0.87	0.19	0.49	1.25	<0.001
Direct effect (SESS-6 → EAT-26)	0.52	0.18	0.16	0.88	0.005
Indirect effect (SESS-6 → BCQ → EAT-26)	0.35	0.11	0.16	0.59	^b^
Direct effect (SESS-6 → BCQ)	0.42	0.10	0.23	0.62	<0.001
Direct effect (BCQ → EAT-26)	0.83	0.13	0.58	1.08	<0.001

^a^ Adjusted for age, BMI, and gender covariates. ^b^ No *p*-value is calculated for the indirect effect. The indirect effect is deemed significant because the confidence interval does not include zero. Sobel’s test further confirmed the statistical significance of this indirect effect. Note. See Note under Table 2. The Mediator is BCQ.

**Table 4:** Sequential mediation analysis (SESS-6 → DAIS-CS → BCQ → EAT-26).

Effect ^a^, Variables	B	se	LLCI	ULCI	*p*-Value
Total effect (SESS-6 → EAT-26)	0.87	0.19	0.50	1.25	<0.001
Direct effect (SESS-6 → EAT-26)	0.47	0.20	0.07	0.87	0.021
Total indirect effect (SESS-6 → EAT-26)	0.40	0.14	0.14	0.70	^b^
**Indirect effect of SESS-6 on EAT-26 serially mediated via DAIS-CS then BCQ (SESS-6 → DAIS-CS → BCQ → EAT-26)**	**0.21**	**0.06**	**0.10**	**0.34**	**^b^**
Indirect effect of SESS-6 on EAT-26 through DAIS-CS; accounting for BCQ (SESS-6 → DAIS-CS → EAT-26)	0.06	0.12	−0.17	0.30	
Direct effect (SESS-6 → DAIS-CS)	2.63	0.33	1.98	3.27	<0.01
Direct effect (DAIS-CS → EAT-26)	0.02	0.04	−0.06	0.10	0.5667
Indirect effect of SESS-6 on EAT-26 through BCQ; accounting for DAIS-CS (SESS-6 → BCQ → EAT-26)	0.13	0.09	−0.04	0.34	
Direct effect (SESS-6 → BCQ)	0.16	0.10	−0.04	0.37	0.118
Direct effect (BCQ → EAT-26)	0.81	0.13	0.54	1.07	<0.001

^a^ Adjusted for age, BMI, and gender. ^b^ No *p*-value is calculated for the indirect effect. The indirect effect is deemed significant because the confidence interval does not include zero. Sobel’s test further confirmed the statistical significance of this indirect effect. Note. See Note under Table 2. The mediators are serially DAIS-CS and BCQ. Results in bold highlight the results of our main analysis, which most closely support our hypothesized sequence.

## Data Availability

The data that support this study’s findings are available from the corresponding author upon reasonable request.

## Supplementary Materials

The following supporting information can be downloaded at: https://www.mdpi.com/article/10.3390/bs16030434/s1.

## Abbreviations

The following abbreviations are used in this manuscript:

*B*	Unstandardized regression coefficient
BCQ	Body Checking Questionnaire
BCQ (OA)	Body Checking Questionnaire Overall Appearance (subfactor)
BMI	Body Mass Index
CI	Confidence Interval
DAIS	Difficulty Accessing Internal States
DAIS-CS	Difficulty Accessing Internal States-Composite Scale
DEP	Disordered Eating Patterns
EAT-26	Eating Attitudes Test-26
IMC	Instructional Manipulation Check
LLCI	Lower Limit of Confidence Interval
OA	Overall Appearance
OCD	Obsessive Compulsive Disorder
SE	Standard error
SESS-6	Six-item State Self-Esteem Scale-6
SPIS	Seeking Proxies for Internal States
SPISI	Seeking Proxies for Internal States Inventory
TAS-20	Toronto Alexithymia Scale
ULCI	Upper limit of confidence interval
