# Supplementary material for: From Self-Esteem to Symptoms: A Potential Role for Difficulties Accessing Internal States and Body-Checking Behavior in Disordered Eating Patterns
Source: Behav Sci (Basel). 2026 Mar 17;16(3):434. doi: 10.3390/bs16030434 (PMC13024392; doi:10.3390/bs16030434)
Supplement: Supplementary file 1 [file behavsci-16-00434-s001.zip › behavsci-4115644-supplementary.pdf]

Supplementary Materials S1

Pre-Analysis Assumption Checks

Model assumptions were examined prior to analysis, and all models satisfied ordinary least squares assumptions. Linearity and homoscedasticity were verified through inspection of standardized residuals plotted against predicted values, showing no systematic deviations. Residuals were normally distributed based on Shapiro–Wilk tests ($p < .001$). Though Q-Q Plots showed one outlier, this was justifiably retained as multicollinearity diagnostics indicated no concerns (VIFs < 2.5; tolerance > .40), and Cook's Distance values were all below 1.0. The Durbin–Watson statistic (1.8) confirmed independence of errors. As PROCESS uses 5,000 bootstrap samples, it provides bias-corrected confidence intervals robust to assumption violations.

Supplementary Materials S2

In Figure 2 (Conceptual Adaptation of the SPIS Model to Disordered Eating Patterns), we illustrate a conceptual model which extends the SPIS model to the field of disordered eating, with self-esteem as the internal state. The following statements describe the elements in the model, with the numbers in brackets denoting the corresponding element in the figure.

[1] A person wishes to answer a question involving their sense of self-esteem, (e.g., am I a worthwhile person?). [2] The person attempts to access this internal state to answer their question. [3] If the answer is clear enough, this process terminates. [4] In the context of low self-esteem, [5] DAIS are more likely, including diminished access to the internal state of self-esteem. [6] In accordance with the SPIS model, the lack of a clear answer leads the person to seek a proxy with which to gauge their self-esteem. For people with low self-esteem (and characteristics such as low body satisfaction or high appearance valuation), body image may be selected as a proxy, and [7] body-checking executed to collect data on this proxy. [8] Body-checking is further coupled with disordered eating behaviors in an effort to control body appearance (the selected proxy), a sequence that, based on extant literature, may be more likely to occur in the presence of an internalized lean-body model.

Critically, while DEP influences the proxy estimated by body-checking, DAIS persist, and therefore the answer to the original question on self-esteem remains unclear, even after disordered eating behavior occurs. While presumably aiming to modify the proxy (body appearance), DEP does not alter the core self-esteem, ameliorate DAIS, alter dominant cognitive schemas, or attenuate inflexible interpretation modifiers. Even in the case that DEP leads body-checking to promote a temporary elevation of self-esteem (e.g., due to a better "fit" to the lean-body model), the unclear answers, coupled with interpretation modifiers (e.g., perfectionism), are likely to maintain the dysfunctional cycle.
